# Targeted inactivation of *Salmonella* Agona metabolic genes by group II introns and *in vivo* assessment of pathogenicity and anti-tumour activity in mouse model

**DOI:** 10.7717/peerj.5989

**Published:** 2019-01-16

**Authors:** Chin Piaw Gwee, Chai Hoon Khoo, Swee Keong Yeap, Geok Chin Tan, Yoke Kqueen Cheah

**Affiliations:** 1 Department of Biomedical Science, Faculty of Medicine and Health Sciences, Universiti Putra Malaysia, Serdang, Selangor, Malaysia; 2 China-ASEAN College of Marine Sciences, Xiamen University Malaysia, Selangor, Malaysia; 3 Department of Pathology, Faculty of Medicine, Universiti Kebangsaan Malaysia, Cheras, Kuala Lumpur, Malaysia

**Keywords:** Attenuated *Salmonella* Agona, Group II intron, LeuB gene, ArgD gene, Anti-tumour therapy, Colorectal cancer

## Abstract

The fight against cancer has been a never-ending battle. Limitations of conventional therapies include lack of selectivity, poor penetration and highly toxic to the host. Using genetically modified bacteria as a tumour therapy agent has gained the interest of scientist from the past few decades. Low virulence and highly tolerability of *Salmonella* spp. in animals and humans make it as the most studied pathogen with regards to anti-tumour therapy. The present study aims to construct a genetically modified *S.* Agona auxotroph as an anti-tumour agent. *LeuB* and *ArgD* metabolic genes in Δ*SopB*Δ*SopD* double knockout *S.* Agona were successfully knocked out using a Targetron gene knockout system. The knockout was confirmed by colony PCR and the strains were characterized *in vitro* and *in vivo*. The knockout of metabolic genes causes significant growth defect in M9 minimal media. Quadruple knockout Δ*SopB*Δ*SopD*Δ*LeuB*Δ*ArgD* (BDLA) exhibited lowest virulence among all of the strains in all parameters including bacterial load, immunity profile and histopathology studies. *In vivo* anti-tumour study on colorectal tumour bearing-BALB/c mice revealed that all strains of *S.* Agona were able to suppress the growth of the large solid tumour as compared with negative control and Δ*LeuB*Δ*ArgD* (LA) and BDLA auxotroph showed better efficacy. Interestingly, higher level of tumour growth suppression was noticed in large tumour. However, multiple administration of bacteria dosage did not increase the tumour suppression efficacy. In this study, the virulence of BDLA knockout strain was slightly reduced and tumour growth suppression efficacy was successfully enhanced, which provide a valuable starting point for the development of *S.* Agona as anti-tumour agent.

## Introduction

Cancer is one of the leading causes of morbidity and mortality worldwide. Conventional anti-cancer therapies often encounter significant side effects and fail to achieve complete tumour remission. Heterogeneous tumour microenvironment, including gradients in chemical concentration and tissue hypoxia make it particularly resistant to systemic treatment (Cairns, Papandreou & Denko, 2006; Klemm & Joyce, 2014). These standard therapies do not target tumour tissue specifically and do not successfully penetrate deep into tumour tissue (Jain, 1998; Tannock et al., 2002), which ultimately leads to loss of local control and tumour recurrence (Davis & Tannock, 2002). A new paradigm for cancer drug development is therefore urgently needed.

Certain live, attenuated non-pathogenic bacteria such as *Salmonella, Bifidobacterium, Clostridium, and Listeria* possess unique features to overcome many of the limitations of chemotherapy (Pawelek, Low & Bermudes, 2003; Morrissey, O’Sullivan & Tangney, 2010; Taniguchi et al., 2010). These bacteria are mostly motile and able to penetrate into tumour tissue which has low oxygen level thus overcoming the limitations of radiotherapy and chemotherapy (St Jean, Zhang & Forbes, 2008; Lee, 2012). Although several bacterial species have been reported as potential anti-cancer agents, most of the current approaches have been focused on *Salmonella* strains.

*Salmonella* characteristics such as motility, propagation control with antibiotics, genetic stability, environmental sensing, native cytotoxicity, low cost of production and safety make it a suitable choice as anti-cancer agent (Chorobik et al., 2013). *Salmonella* is able to produce certain virulence factors leading to cytotoxicity and induce innate immunity to target tumours which helps in further tumour regression (Lee, Wu & Shiau, 2008; Lee, 2012; Kaimala et al., 2014). Furthermore, *Salmonella* can be delivered in low dose followed by proliferation to an effective dose within the target tumour (Forbes et al., 2003). Furthermore, since *Salmonella* is a facultative anaerobe, it is able to colonize large and small tumours and even accumulate within metastases after systemic administration (Leschner & Weiss, 2010; Yam et al., 2010). It was reported that *Salmonella* auxotroph with the deletion of *msb*B and *pur*I gene would preferentially replicate within tumours when inject into tumor-bearing mice, showing tumour to normal tissue ratio exceeding 1000:1. The accumulation of *Salmonella* in tumour is accompanied by delay in tumour growth (Pawelek, Low & Bermudes, 1997).

Altogether, bacterial anticancer therapy approach has made great progress in past decades. Despite the advantages and potential for live bacteria as anti-tumour agent, it is clear that in many cases fundamental studies are needed to address issues such as side-effects and also to improve the efficacy of the system. Employing powerful genetic engineering tools it is desirable to engineer strains to reduce toxicity in host and enhance therapeutic activity. In this study, group II intron technology has been utilized to silence the metabolic genes in *Salmonella* Agona. The genetically modified *S.* Agona leucine and arginine auxotroph was expected to exhibit lower virulence and exert higher level of anti-tumour activity. The construction of genetically modified *S.* Agona followed by characterization *in vitro* and *in vivo* is presented in this study.

## Materials and Methods

### Bacterial strains, growth and storage conditions

A food source strain *S.* Agona (Ag1) and a genetically modified Ag 1 were used in this study. The food source strain *S.* Agona was recovered from indigenous vegetables in Selangor state, Malaysia (Khoo et al., 2009). The genetically modified Ag1 was constructed in previous work with double mutations in *sopB* and *sopD* genes, termed as Δ*sopB*Δ*sopD* double knockout (Khoo et al., 2015). This particular Δ*sopB*Δ*sopD* double knockout was used in this study for further genetic engineering work to further reduce its pathogenicity. The bacteria was cultured in Nutrient Broth or Nutrient agar plate (Merck KGaA, Darmstadt, Germany) at 37 °C and stock stored in 15% glycerol at −80 °C.

### Cancer cell line and growth conditions

*Mus musculus*, mouse colon carcinoma cell line, CT-26 was obtained from American Type Culture Collection (ATCC), USA, and cultured in Roswell Park Memorial Institute 1640 (RPMI 1640) (ScienCell, San Diego, CA, USA) complete medium supplemented with 10% fetal bovine serum (Sigma-Aldrich, Darmstadt, Germany), 1% v/v penicillin/streptomycin (Nacalai Tesque, Japan) and 2% of L-glutamine (Gibco Life Technologies, São Paulo, Brazil) in a humidified incubator with 5% CO_2_. Cell line was detached from the culture flask using a 0.25% trypsin-EDTA solution and re-suspended as monolayer cell suspension in RPMI 1640 culture medium.

### Group II intron targeted gene disruption

*Salmonella* Agona *leuB* and *argD* metabolic genes were amplified using polymerase chain reaction (PCR) in the present study followed by gene disruption using Targetron Gene knockout System (Sigma-Aldrich, Darmstadt, Germany). A computer algorithm at Sigma-Aldrich Targetron design website (http://www.Sigma-aldrich.com/targetronaccess) was used to identify the potential L1.LtrB insertion sites. Three primers were designed to modify the RNA portion of intron template which base pair optimally to corresponding insertion sites, referred to as IBS, EBS1d, EBS2. The designed primers are listed in . A splicing-by-overlap-extension (SOE) PCR reaction which retargets the RNA portion of the intron by primers-mediated mutation was performed according to the manufacturer’s protocol with slightly modification. The purified 350 bp PCR containing modified intron portion was then digested with *Hind*III (1U) and *Bae*GI (0.5U) (Sigma-Aldrich, Darmstadt, Germany) followed by ligation into the pACD4-C intron donor chloramphenicol-resistant linear vector (2 ng) (Sigma-Aldrich, Darmstadt, Germany). The ligation reaction was performed using Quick-link T4 Ligation Kit (Sigma-Aldrich, Darmstadt, Germany) according to the manufacturer’s instruction. The ligated product was first transformed into *E.coli* QIAGEN EZ competent cells due to low transformation competency of *S.* Agona strain. The pooled plasmid from *E.* coli was then co-transformed with plasmid pAR1219 which expressed T7 RNA polymerase into *S.* Agona via electroporation. The intron insertion in the transformant was identified by colony PCR using gene specific primers which amplifies the entire intron inserted target gene. To adapt the system for multiple gene knockouts, the first constructed *leuB* knockout was applied to sequentially mutate the second *argD* metabolic gene for the construction of Δ*leuB*Δ*argD* double knockouts. The Δ*sopB*Δ*sopD* double knockout was subjected to the same experiment as mentioned above to construct the quadruple knockout Δ*sopB*Δ*sopD*Δ*leuB*Δ*argD*.

### *In vitro* growth assessment of knockout strains

Growth curve analysis of wild-type *S.* Agona (W), double knockout Δ*leuB* Δ*argD* (LA), double knockout Δ*sopB* Δ*sopD* (BD) and quadruple knockout Δ*sopB* Δ*sopD* Δ*leuB* Δ*argD* (BDLA) was carried out in M9 minimal media. Each overnight culture in Nutrient broth was washed twice with phosphate-buffered saline (PBS) prior to inoculation in M9 minimal media. M9 minimal media was prepared according to recipe describe by Sambrook (Sambrook & Russell, 2001). Overnight bacterial culture was then inoculated in M9 minimal media at 1:100 ratio. Double knockout LA and quadruple knockout BDLA was cultured in M9 minimal media supplemented with leucine and arginine separately, and both leucine and arginine together in respective flasks. Samples were incubated with shaking (200 rpm) at 37 °C. Bacterial growth was recorded for a total period of 12 h by measuring the OD_600_ of the cultures at intervals of 1 h.

### Invasion assay

The CT-26 cells at number of 1 × 10^5^ were seeded in 96-well plate for 24 h at 37 °C. Cell lines were washed twice with sterile PBS prior to the infection with different *Salmonella* strains. *Salmonella* strains in late-log phase growth were diluted in 100 µl of RPMI media and added at the 100 multiplicity of infection (MOI). After infection, cells were washed three times with PBS and then treated with 100 µg/mL of gentamicin (Bio-Basic, Markham, Ontario, Canada) to kill the external bacteria, for 1 h at 37 °C. After the gentamicin incubation, the cells were washed for three times again and then incubated with 0.1% Triton X-100 (Merck, Darmstadt, Germany) for 10 min at 37 °C. The number of internalized bacteria was determined by plating ten-fold serial dilutions of the cell lysates on xylose lysine deoxycholate (XLD) agar plates.

### *In vivo* toxicity profile of attenuated *Salmonella*

### Survival test analysis

Mice used in this study were purchased from a local supplier (Saintifi Enterprise, Kuala Lumpur, Malaysia) and adapted for one week prior to the experiment (see ‘Ethics Statement’ below). All mice were maintained in an enriched environment and in accordance with institutional animal care and guidelines. Consent for experiments involving animal was obtained from the appropriate University Putra Malaysia (UPM) Ethics Committee. Six-to-eight week old male, BALB/c mice were randomly divided into five groups and treated with W, BD, LA, BDLA and PBS as negative control. The inoculums were administered intraperitoneally with different concentrations of bacteria ranging from 10^1^ to 10^7^ cfu in 100 µl PBS. Animal deaths were recorded for 30 days.

### Ethics statement

All animal experiments conformed to the Institutional Animal Care and Use Committee of Universiti Putra Malaysia using the protocol UPM/IACUC/AUP-R056/2013.

### Evaluation of potential toxicity of bacterial infection

Six-to-8 week-old male, BALB/c mice were divided into five groups and treated with W, LA, BD, and BDLA at concentration of 10^4^ cfu in 100 µl of PBS. PBS was used as negative control. Mice were sacrificed at day-1, day-3, day-10, day-21 and day-36. The potential side effects and toxicities induced by the treatments were evaluated in the aspect of bacterial distribution, histopathology and full blood count analysis.

### Analysis of bacterial distribution

The isolation and titration of bacteria from mice were performed as described with slight modification (Jia et al., 2007). Briefly, five organs include liver, spleen, kidney, intestine, and lung were removed aseptically and homogenized with PBS at a ratio of 10:1 (PBS volume (mL): tissue weight (g)). The homogenates were then serial diluted in ten-fold dilution, and plated onto XLD agar and incubated overnight at 37 °C. The titer of bacteria in each organ was determined on the next day by counting colonies and dividing them by weight of tissue (c.f.u/tissue (g)).

### Histological analysis

Portion of liver, spleen, lung, kidney and intestine were fixed immediately with 10% paraformaldehyde/0.1 M phosphate buffer (PB) with fixative volume of 15–20 times greater than tissue volume. The subsequent embedding and staining processes were proceeding according to standard histological procedures. Inflammation and infectious foci of the tissues were observed under a light microscope to evaluate the severity of inflammation induced by bacteria.

### Full blood count analysis

Blood samples were collected from mice at day-1, 3, 10, 21, 36 days post infection. Blood samples were collected through cardiac puncture and were stored immediately in EDTA blood collection tubes. The level of neutrophils, lymphocytes and white blood cells were determined subsequently by Haematology and Clinical Biochemistry Laboratory, Faculty of Veterinary, Universiti Putra Malaysia.

### *In vivo* tumour growth suppression

Tumour growth inhibition assay was conducted in mice with large and small tumour model separately and each tumour model was subjected to single and multiple administrations of the treatments. For tumour implantation, 6-to 8-week-old male, BALB/c mice were implanted subcutaneously on the mid-right flank with CT-26 colon carcinoma cells at concentration of 10^6^ cells in 100 µl of PBS. Tumour was allowed to grow for 2 weeks before treatment. Mice were examined daily until the tumour size was approximately 250 mm^3^ and 450 mm^3^ for small and large tumour respectively. Different *Salmonella* strains, W, BD, LA and BDLA were administered intraperitoneally at concentration of 10^4^ cfu in 100 µl of PBS. Anti-tumour activity of the treatments was evaluated by measuring tumour size every 2 days. Tumours were measured individually with a digital caliper. Tumour volumes were determined by the formula: tumour volume = length × width^2^ × 0.52 (Yoon et al., 2011). The experiments was repeated as above experimental groups, the mice were re-treated 1 week after the first treatment and were followed up to 30 days for tumour size analysis.

### Statistical analysis

Statistical significance of experimental results was determined by Student’s *T*-test to compare the differences among two groups. For multiple group comparison, one-way ANOVA analysis of variance was performed followed by post-hoc analysis. All data are expressed as the mean ± SEM; a *p*-value less than 0.05 was considered to be statistically significant. The statistical analysis was performed using SPSS software (version 16.0; SPSS, Chicago, IL, USA).

## Results

### *leuB* and *argD* metabolic gene knockout using group II intron technology

**Figure 1:**
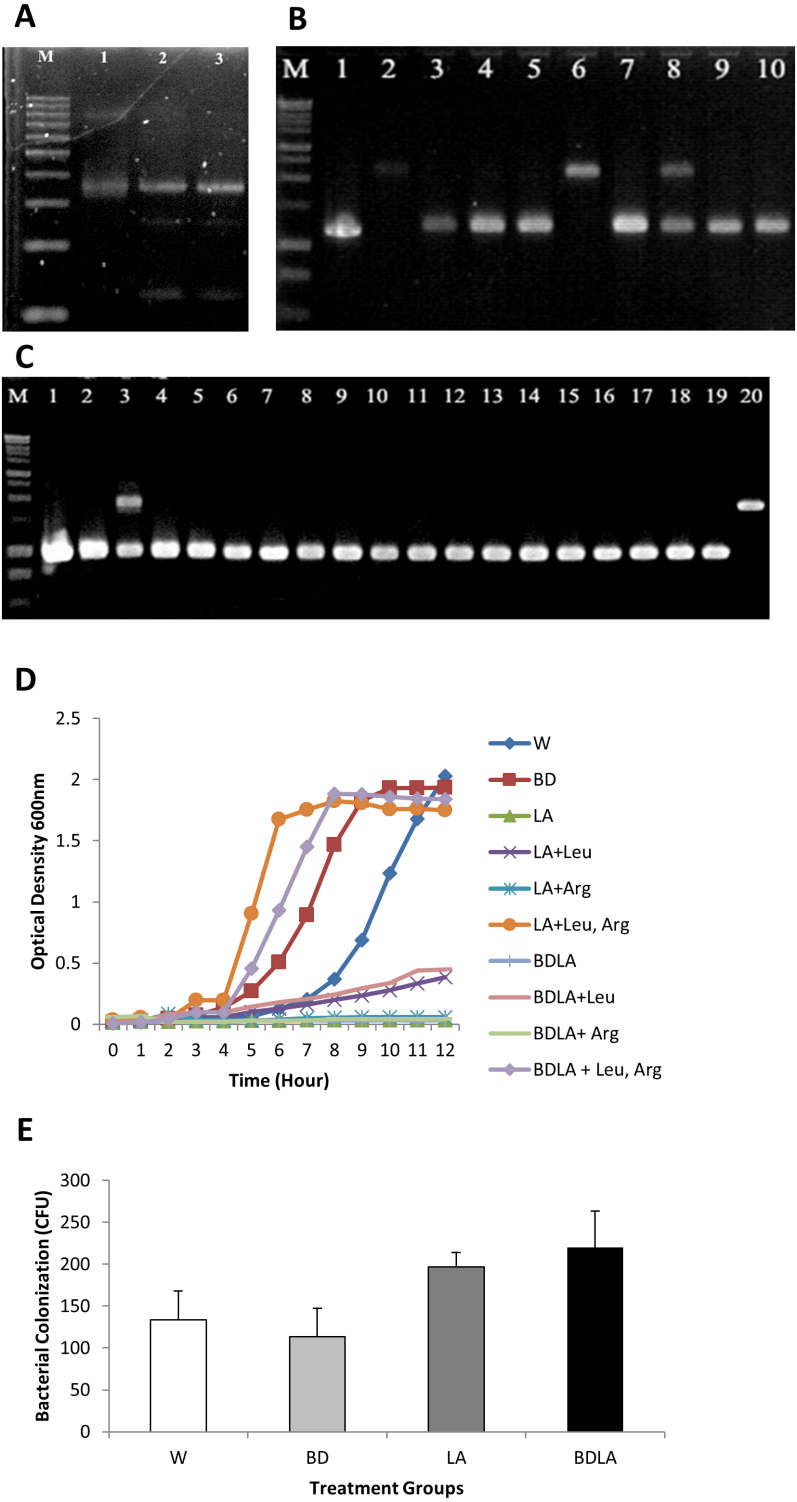
Representative of PCR amplification showing successful retargeted intron in *S*. Agona (A), colony PCR to confirm *leuB* and *argD* knockout (B) & (C), growth kinetics (D) and *in vitro* invasion activity (E). Successful retargeted intron in *leuB* and *argD* gene in *S.* Agona. Splicing overlap extension polymerase chain reaction (SOE PCR) was performed to modify the RNA portion of the intron, which resulted in a 350 bp retargeted intron. Lane 1: Retargeted intron targeting *E.coli lacZ* gene as positive control; Lane 2: Retargeted intron targeting *leuB* gene in *S.* Agona. Lane 3: Retargeted intron targeting *argD* gene in *S.* Agona. (B) & (C) Intron insertion into targeted gene was identified by colony PCR using gene-specific primers. The clones with successful targetron insertion showed a 1,000 bp increase in size as compared with wild-type *S.* Agona. (B) Intron insertion in *leuB* gene of *S.* Agona. Lane 2 and 6: PCR product with targetron insertion (2,043 bp) in pure *Salmonella* clone. (C) Intron insertion in *argD* gene of *S.* Agona. Lane 20: PCR product of *argD* with targetron insertion (1,887 bp) in pure *Salmonella* clone. (D) Growth kinetics of wild-type *S.* Agona, double knockout Δ*sopB*Δ*sopD*, double knockout Δ*leuB*Δ*argD*, and quadruple knockout Δ*sopB*Δ*sopD* Δ*leuB*Δ*argD* in M9 minimal media with different conditions. Growth of leucine and arginine auxotroph was defective in M9 minimal media and the growth was fully reverse with external supplement of the missing biomass. (E) *In vitro* invasion activity of wild-type and genetically engineered *Salmonella* on CT-26 cell line. CT-26 was treated with gentamicine and Triton X-100 after incubation with *Salmonella* in order to determine the number of intracellular *Salmonella* Agona cells. No difference of intracellualr bacterial counts was observed, indicating that invasiness of genetically-modified strains is not affected.

Group II intron technology was utilized to construct genetically modified *S.* Agona. The potential targets sites for *leuB* (9 sites) and *argD* (5 sites) were predicted by Sigma-Aldrich Targetron. Targetron integration was successful for the 6th design for *leuB* and 2nd design for *argD* (Fig. S1). Splicing overlap extension polymerase chain reaction (SOE PCR) was then performed to modify the intron sequence. As shown in Fig. 1A, SOE PCR gave a 350 bp of retargeted intron for *leuB* and *argD* gene which is necessary to cause gene disruption. The knockout mutant strains were confirmed by colony PCR using gene-specific primers to flank their respectively inserted intron. The results (Figs. 1B & 1C) show that the intron has been successfully inserted resulting in a significantly increased PCR product sizes approximately 1,000 bp as compared with wild-type *S.* Agona. Analysis of 92 *S.* Agona colonies for *leuB* insertion showed intron insertion occurred at a frequency of 15% where 14 colonies contained intron-inserted gene, that produced expected 2,043 bp PCR product (Fig. 1B). For *argD* gene, three out of 92 colonies analysed showed gene insertions, indicating gene targeting at the frequency of 3%, which produced a PCR product of around 1,887 bp (Fig. 1C). Stability of intron insertion and purity of clone isolated was evaluated by passaging the bacterial culture in medium for 30 days consecutively and no reversion to wild-type was observed (Fig. S2A). The inserted intron were further verified by PCR amplified and sequenced using the gene-specific primers (Figs. S2B & S2C).

### Growth of auxotrophic mutant is defective but invasiveness is not affected *in vitro*

The *in vitro* phenotypic characteristic of genetically modified *S.* Agona was assessed by evaluation of growth in M9 minimal media. Evaluation of growth kinetics revealed that the genetic engineered mutant strains that carried the *leuB* and *argD* insertional mutations in *S.* Agona caused remarkable changes in growth characteristics. Double knockout Δ*leuB* Δ*argD* (LA) and quadruple knockout Δ*sopB* Δ*sopD* Δ*leuB* Δ*argD* (BDLA) were unable to grow in M9 minimal media throughout 12 h. Both of the metabolic gene knockout strains were able to grow partially in M9 minimal media supplemented with leucine only reaching final cell densities OD_600_ ∼ 0.4 compared with OD_600_ ∼ 2.0 for the parental strain after incubation period of 12 h. However, both genetically modified LA and BDLA strains were still unable to grow in M9 minimal media supplemented with arginine only. Growth inhibition of LA and BDLA mutant strains were able to be fully reversed with the supplementation of both leucine and arginine in M9 minimal media. The corresponding cultures reached final cell densities (OD_600_ ∼ 1.9) that were similar to the parental strain *S.* Agona. Growth ability of double knockout Δ*sopB* Δ*sopD* (BD) is not affected as it reaches similar cell density as the parental strain throughout 12 h of incubation period. *Salmonella* is an intracellular bacteria and invasion is essential to cause the infection. We next examined the invasiveness of these modified *S.* Agona strains and found no significant difference in the recovered bacterial colony forming unit (Fig. 1E). Together, these data indicate that the modification of *leuB* and *argD* genes affect bacterial growth without altering its invasion property.

### BDLA mutant replicate slower in internal organ and induced lower inflammatory response

Our previous study has shown that *sopB* and *sopD* genes are not required for full pathogenicity in *C. elegans* model as there is no virulence difference observed between wild-type and mutant strains (Khoo et al., 2015). In order to provide a better understanding of these virulence and the link with metabolic genes in *Salmonella*, the mutants *S.* Agona were subjected to pathogenicity analysis by following mice survivability, bacterial colonization and histolopathological analysis.

**Figure 2:**
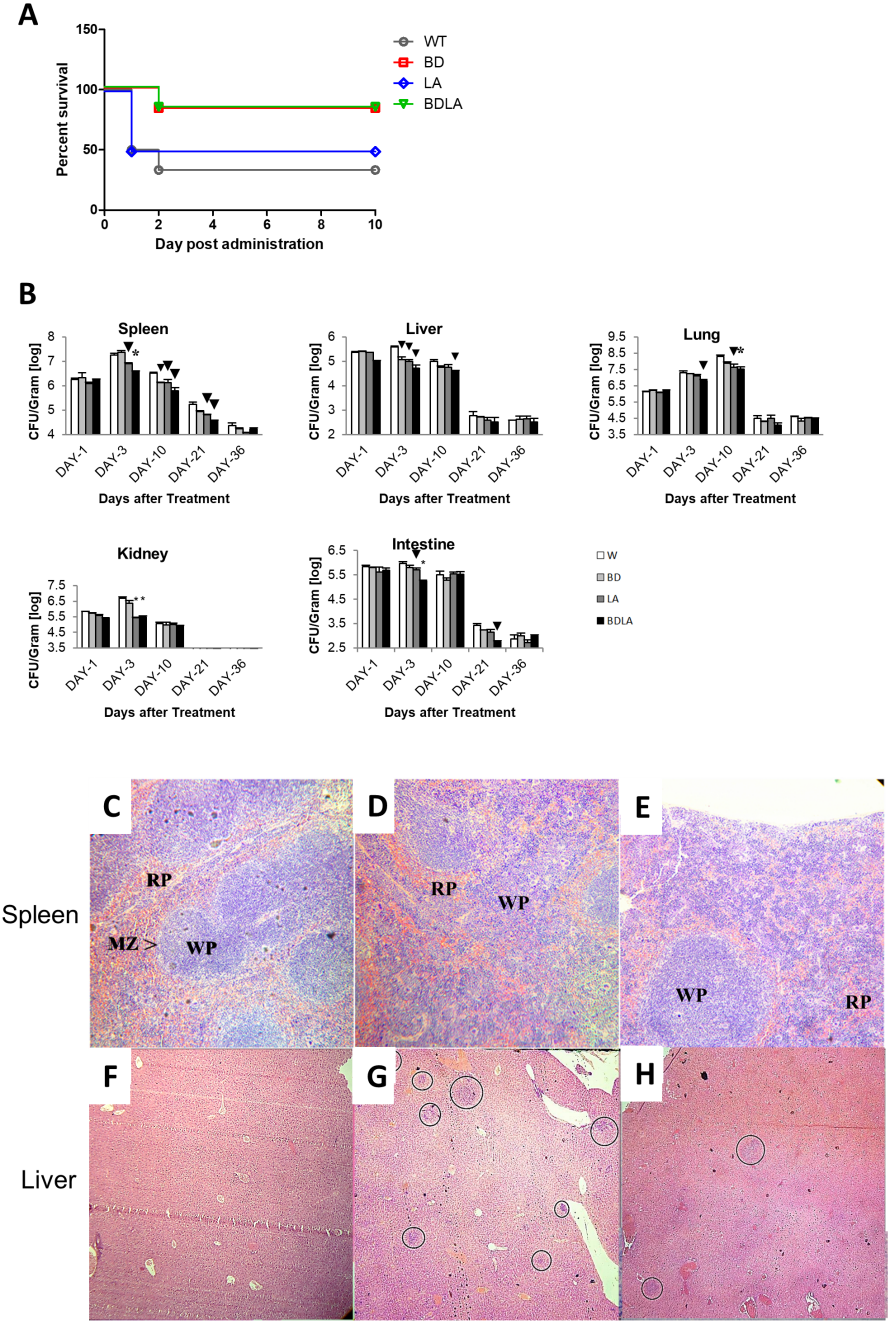
Survival curve (A) *in vivo* colonization of microbe in vital organs of tumour free mice (B) and histopathology findings (C). (A): Survival curve of mice post intraperitoneal administration of different *S.* Agona strains at the concentration range of 10^3^ cfu to 10^7^ cfu. Dosage at 10^4^/cfu contributed to the difference on mice survivability between all of the treated groups. The survival rate for W is 33.33%, 83.33% for BD, 50% for LD and 83.33% for BDLA. Overall, slightly improvement of mice survivability was noticed after genetic modification. (B) Colonization of spleen, liver, lung, kidney and intestine in tumour-free mice by wild-type and mutants *S.* Agona after intraperitoneal administration. Tumour-free mice were administrated with different *Salmonella* as indicated. Bacteria replication in each vital organ was determined at 1, 3, 10, 21 and 36 days post administration. Each bar represents the mean ± SEM of four mice per group. (▾) *P* < 0.05; * *P* < 0.01 when compared with W group. (C–H) Representative histopathology analyses of spleen (H&E, 100X) and liver (H&E, 40X) sections excised from the PBS control,wild-type and BDLA treated tumour-free mouse after 10 days of intraperitoneal injection. Disruption of splenic architecture and inflammation is more severe in (D). A higher number of infection foci were observed in (G).

To investigate if the genetically modified *Salmonella* strains could possibly lead to lower severity and lethality, BALB/c mice were infected with 1 ×10^3^ to 1 ×10^7^ cfu of wild-type and genetically modified strains and monitored for survival over 30 days. Wild-type and genetically modified *S.* Agona-infected mice at the dosage of 1 ×10^5^ −1 ×10^7^/cfu died on the next day after infection. However, all of the mice survived up to 30 days with the lower dosage 10^3^/cfu for each of the treatment groups and dosage at 10^4^/cfu contributed to the difference in mice survivability between all of the treated groups. As shown in Fig. 2A, the survival rate for wild-type (WT) infected mice is 33.33%, 83.33% for BD, 50% for LD and 83.33% for BDLA. Overall, slightly improvement of mice survivability was noted after genetic modification.

To establish whether the bacteremia resulted in colonization of internal organs, we assayed the number of bacteria in spleen, liver, lung, intestine and kidney at five different time points. Systemic infection could be detected starting from day 1 post-infection in both the wild-type and genetically modified *S.* Agona-infected mice as indicated by the presence of bacteria in all vital organs tested (Fig. 2B). The bacterial load in the examined tissues increased on day-3 post-infection and started to decline from day 10 post-infection, except for the lung tissue which only started to reduce by day 21 post-infection (Fig. 2B). Remarkably, BDLA-infected mice demonstrated the lowest bacterial load amongst all the genetically modified strains in spleen (∼5-fold lower than WT on day-3 and day-10 post-infection) and liver (7.4-fold and 2.5-fold lower than WT on day-3 and day-10 post-infection respectively).

Histology was performed to examine the inflammatory response of spleen and liver to *Salmonella* infection. The spleen from the PBS-control mouse **s**howed normal architechture of splenocytes, with clear distinction between the red (RP) and white pulp (WP) (Fig. 2C). As for the mouse challenged with wild-type *S.* Agona (Fig. 2D), the white pulps are fused. Besides that, marginal zone (MZ) were not clearly observed and number of extramedullary hematopoiesis was increased. However, disruption of normal splenic architechture and inflammation was less severe in the mouse infected with BDLA mutant *S.* Agona (Fig. 2E) as compared to wild-type. We next quantified the number of infection foci within liver to assess the severity of inflammation. As shown in Fig. 2F, liver from the mouse injected with PBS showed normal histology and no infection foci were seen. Liver from the mouse challenged with wild-type *S.* Agona showed higher number of infection foci per field (Fig. 2G) compared to liver of mouse infected with BDLA mutant *S.* Agona (Fig. 2H). This phenomenon indicates that BDLA may have lower pathogenicity.

**Figure 3:**
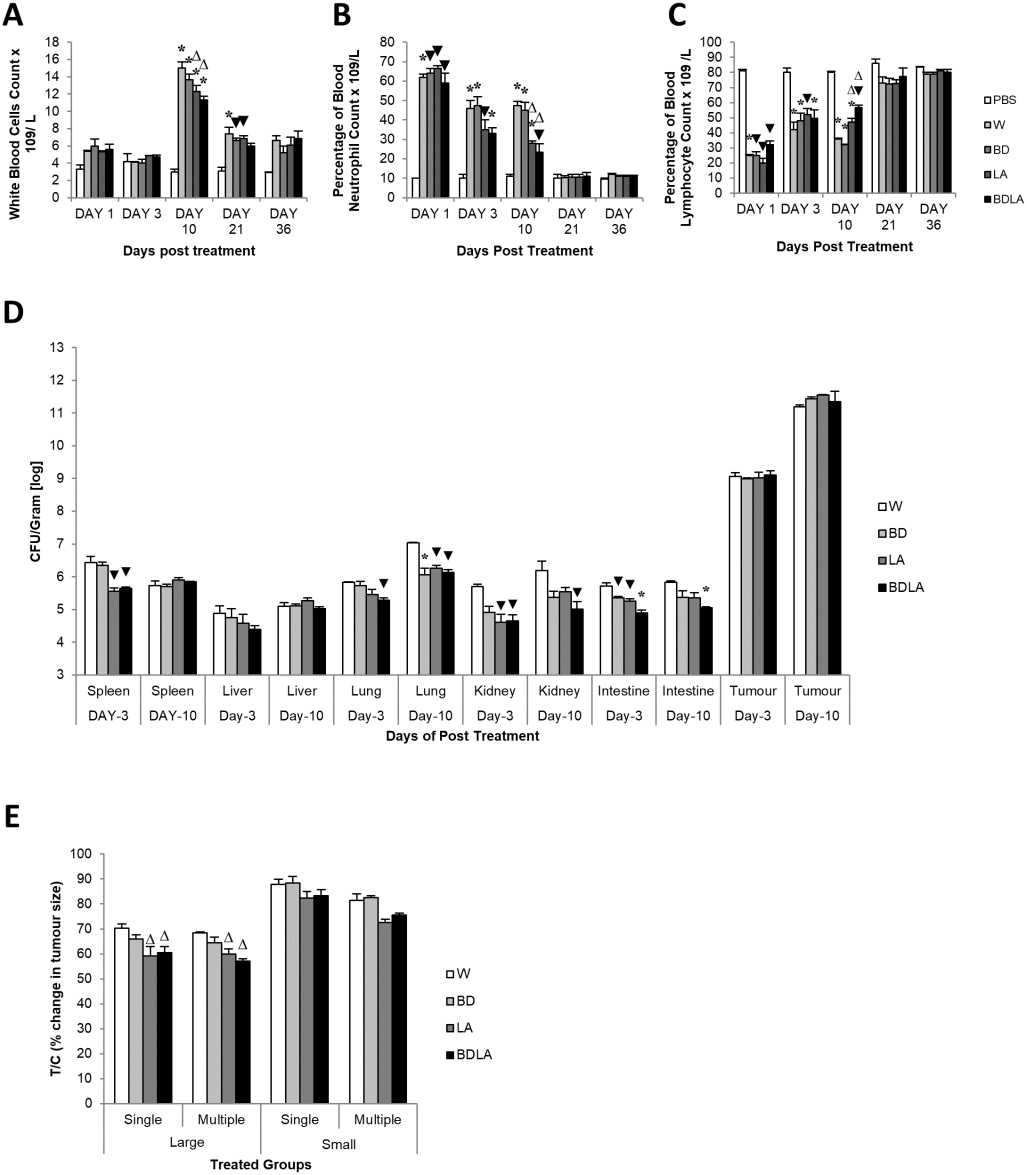
Blood parameter profiles (A, B, and C), *in vivo* colonization of vital organs in tumour bearing mice (D) and percentage of changes in tumour size (E). (A) Total white bood cell concentration, (B) percentage of blood neutrophile and (C) percentage of blood lymphocyte in tumour-free mice. Tumour-free mice were administrated with *Salmonella* and blood was collected post administration at indicated time point. Each bar represents the mean ± SEM of four mice per group. (*) *P* < 0.01 and (▾) *P* < 0.05 when compared with PBS control; (Δ) *P* < 0.05 when compared with W group. (D) Colonization of spleen, liver, lung, kidney and intestine in CT-26 tumour-bearing mice by wild type and mutants *S.* Agona after intraperitoneal administration. Tumour-bearing mice were administrated with different *Salmonella* as indicated. Bacteria replication in each vital organ was determined at day-3 and day-10 post administration. Each bar represents the mean ± SEM of four mice per group. (▾) *P* < 0.05; * *P* < 0.01 when compared with W group. (E) The percentage change in tumour size of the treated related to the control group (T/C) in large and small tumour model with single and multiple administration of treatments. Each bar represents the mean ± SEM of four mice per group. (Δ) < 0.05 when compared with W treatment group. LA and BDLA showed slightly better efficacy in large but not small tumour model.

We then assessed the immunological response evoked by wild-type and mutant strains by determining the titer of white blood cell, neutrophil and lymphocyte at 5 different time points. Administration of *S.* Agona into BALB/c mice led to significant increment in total white blood cell concentration at day-10 post-infection as shown in Fig. 3A. The treated group showed significant difference compared with the control group (PBS) on day-10 and day-21 post-infection. At day-10 post-infection, LA and BDLA treated group showed 1.2-fold and 1.3-fold lower level of induction respectively when compared to WT group. *Salmonella* infection also induced a significant increase in neutrophil level in mice on day 1 post-infection and subsequently declined on day-3 post-infection followed by normal level on day-21 post-infection (Fig. 3B). *Salmonella* treated group demonstrated significant difference in neutrophil level compared with PBS control group from day-1 to day-10. LA and BDLA led to modest changes in the increment of neutrophil level, with 1.7-fold and 2-fold lower than WT on day 10 post-infection. Furtherpore, *Salmonella* infection also caused suppression of lymphocytes on day 1 post-infection and subsequently increased on day-3 post-infection (Fig. 3C). Lymphocyte level returned to normal level on day-36 post-infection. *Salmonella* treated groups showed significant difference in lymphocyte level compared to PBS control group from day 1 to day 10 (Fig. 3C). At day-10, treatment with LA and BDLA led to modest suppression of lymphocyte level with 1.3-fold and 1.6-fold lower level as compared to WT group. No significant differences were observed between treated group and control group for the rest of the indicated time points. The results from all the tests indicate that BDLA has the lowest virulence as compared with the others.

### Auxotrophic strain LA and BDLA show higher tumour growth suppression efficacy

In order to evaluate the potential effect of Leu,,Arg auxotrophy of *S.* Agona on tumour growth *in vitro*, CT-26 colon carcinoma-bearing mice were treated with 1 × 10^4^ cfu of mutant strains. The bacteria in liver and tumour were quantified *in vitro* and the number of colony forming units (cfu) per gram of bacteria in tumour was found to be 2 ×10^6^-fold higher than liver, indicating that genetically modified *S.* Agona auxotrophic strain prefer to colonize in tumour tissue (Fig. 3B). Assessment of anti-tumour activity mediated by the genetically-modified strains using T/C% (treated over control tumour volume ratio) revealed a modest decreased in the tumour size compared to WT (Fig. 3C). In large tumour model, LA and BDLA able to show lower T/C% when compared to WT treated group (Fig. 3C) (*W* = 70.36 ± 1.6922; BD = 65.98 ± 1.6636; LA = 59.09 ± 3.806; BDLA = 60.5 ± 2.4758). However, in small tumour model there was no significant tumour growth suppression observed for all the treatments. We also investigated if multiple administration of bacteria dosage could enhance the effectiveness of tumour suppression but found no improvement in the therapy (Fig. 3C). We also examined the side effects of these genetically-modified strains in tumour-bearing mice. The bacterial load in the spleen of tumour-bearing mice was lower compared to tumour-free mice (Fig. S3A). No significant difference was observed in the body weights of the treated and untreated groups (Fig. S4). Additionally, lower systemic neutrophil levels were found in tumour-bearing mice compared to tumour-free mice (Fig. S3). These data suggest that accumulation of *Salmonella* in tumour tissue induced less side effects.

## Discussion

*Salmonella enterica* subsp. *enterica* serovar Agona (abbreviated as *S.* Agona) is a food borne pathogen for human and animal and yet very little information about this bacterium has been reported. To date, there is no genetically modified *S.* Agona reported except triple knockout strain Δ*sopB*Δ*sopD*Δ*PipD* from our previous work (Khoo et al., 2015). This study aimed to construct genetically modified *S.* Agona, a strain which is proven to be less virulent as compared with *S.* Typhimurium, and engineer it as a potent anti-tumour therapeutic agent. Specifically the inactivation of *leuB* and *argD* metabolic genes in the background of the double knockout strain Δ*sopB*Δ*sopD S.* Agona in order to enhance the anti-tumour capacity while at the same time reducing its virulence was examined. Group II intron technology was employed to specifically inactivate the targeted genes. The Sigma-Aldrich Targetron computer algorithm was used to identify the potential targetron insertion sites within the target gene. Previous study reported that retargeted group II intron able to insert specifically on the target gene of *E*. coli at frequency of 0.1–22% (Karberg et al., 2001). A similar intron insertion frequency was seen in our study where the frequency was 3–15% for both targeted genes. Some of the positive colony PCR products contained mixed *Salmonella* clones. This is likely to be due to the heterogeneity in the bacterial progeny, whereby not all bacteria contain the chromosomal disruption by targetron (Chen et al., 2005). Consequently, a single colony would contain some bacteria with targetron-inserted *leuB*/*argD* genes and some bacteria with wild type *leuB*/*argD* genes

To gain some insights on how does these genes affect the *in vitro* phenotypic of the mutant strains, we subjected these mutants to growth and invasion analysis. M9 is a minimal growth medium that provide minimum growth substances, and can be used to select auxotrophs that are not able to produce certain amino acids, hence not able to grow in M9 media. As shown in our study, leucine and arginine auxotrophs did not grow in M9 broth. *S.* Agona *leuB* gene encodes 3-isopropylmalate dehydrogenase enzyme that takes place in the third step of leucine biosynthetic pathway (McCourt & Duggleby, 2006) and inactivation by intron insertion has blocked the leucine biosynthetic pathway. Hence, no leucine metabolites can be produced and the mutant requires external supplementation of leucine in order to survive. Similar finding was also demonstrated in another study (Rajasekaran et al., 2008), where *Brucella abortus* Strain RB51 leucine auxotroph was unable to grow in leucine-deficient condition after the inactivation of its *leuB*. The *argD* gene encodes an enzyme called N-acetylornithine aminotransferase that is involved in fourth step in arginine biosynthetic pathway (Riley & Glansdorff, 1983; Ledwidge & Blanchard, 1999) and the inactivation of this gene is expected to block the synthesis of arginine protein in *S.* Agona. However, inactivation of *argD* appears to be not complete since both LA and BDLA *S.* Agona mutant strains were able to grow partially without supplement of external arginine in M9 minimal media. This observation was not in agreement with study by Ramos et al. (2014) where the *arg* D1000::Tn5 *Erwinia amylovora* mutant was shown to be not able to grow in M9 minimal media. This is quite surprising since we have shown the intron insertion had occurred successfully. We therefore hypothesize that redundancy could play a role and that other non-specific transaminases could replace the activity of N-acetylornithine aminotransferase (Kuo & Stocker, 1969); thus, these mutant strains may be able to grow slowly in M9 minimal media. Inactivation of the virulence genes *sopB* and *sopD*, does not affect the metabolic activity, therefore growth kinetics of double knockout BD was not affected after genetic modification.

*Salmonella* is an intracellular pathogen and invasion is a crucial step during tumour treatment, which allows the bacteria to attack cancer cells (Chorobik et al., 2013). Therefore, evaluation of the invasion capability of genetically modified *S*. Agona is necessary. *leuB* and *argD* gene encodes for the enzyme responsible in the metabolic pathway that produce energy and nutrients for growth and cell division. Thus, *Salmonella* invasiveness is not affected after inactivation of the genes mentioned. The study by Zhao et al. (2006) showed similar finding where *S.* Typhimurium A1-R mutant Leu-Arg auxotroph was able to invade and replicate in HT-29 tumour cells. Other studies have demonstrated that *sopB* and *sopD* contribute to the invasiveness of epithelial cells, where higher number of wild-type *S.* Typhimurium was recovered in gentamycin protection assay (Raffatellu et al., 2005) as compared to *sopB* and *sopD* mutant strain. This report was inconsistent with the finding in the current work since there was no difference in the colony forming unit recovered between wild-type and the double knockout BD. This phenomenon may be due to *sopD* mutant invading the polarized but not the non-polarized T84 colorectal carcinoma cells. Polarized cell lines are more likely to resemble epithelial cells which are normally encountered by *S.* Typhimurium in the intestinal mucosal (Raffatellu et al., 2005). The *in vitro* polarization cell system is required in maintenance of cellular polarity (Criss & Casanova, 2003) which is not available in the present work. Invasiveness of *S.* Agona with *sopB* gene inactivation has not been studied and this is the first report. The inconsistency in the findings reported here and elsewhere are likely to be a result of different methodologies used, hence further investigations are required in order to study the underlying mechanisms.

Infection of *Salmonella* in mouse model closely resembles the characteristic features of human diseases such as typhoid fever and enteritis (Santos et al., 2001). Thus, the mouse model is ideal for *Salmonella* related studies. In our study, we found that the leucine and arginine auxotroph do not prolong the mice survivability. Mice survivability following infection of genetically modified *S.* Agona auxotroph in present study is not in agreement with the findings by Zhao et al. (2004), where all the mice infected with *Salmonella* Typhimurium A1 Leu-Arg auxotroph at concentration of 10^7^/cfu were able to survive up to 10 days after infection. We hypothesize that the attenuated *Salmonella* auxotroph should not be able to survive unless the missing biomass components were provided externally. Endogenous leucine and arginine in the mouse tissues could be freely accessible to *S.* Agona auxotroph leading to their survival, hence causing the observed lethality in mice. This is further evidenced by a publication which demonstrated the ability of *purH* mutant *Salmonella* (an auxotroph for purine) to survive and cause lethal infection in mice (McFarland & Stocker, 1987) since it was estimated that around 1800 molecules of purine could be transported into *Salmonella* cell per second from the host environment (Bumann, 2009). Furthermore, it has been shown that mutant with leucine auxotrophy and mutant with arginine utilization defects were able to present full virulence in the competitive infections, suggesting that the attenuated *Salmonella* is able to access the nutrients from host (Steeb et al., 2013). It is also probable that a different genetic manipulation methodology could attribute to the difference in gene attenuation which would eventually lead to different mouse survivability. Chemical mutagenesis has been shown to be a random process and actual pathways or genes affected are often unknown (Kadonaga & Knowles, 1985). Hence, it may be possible that the chemical mutagenesis method adopted in Zhao et al. study might mutate certain genes (eg. transporters) that accentuate the blocking of leucine and arginine synthesis completely in *S.* Typhimurium A1. This hypothesis was supported by the evidence that different purine mutation produced different outcome on mouse-virulence (McFarland & Stocker, 1987). Our study also showed that mutation on *sopB* and *sopD* were not able to enhance the mice survivability. *sopB,* a gene located on *Salmonella* pathogenicity islands-5 (SPI-5) is responsible for gastroenteritis by increasing the fluid secretion in the intestine of infected host. *sopB* mutant of *S.* Typhimurium and *S.* Dublin blocked their ability to cause gastroenteritis but they were still able to cause diarrhea and death in calves at the higher dosage (Galyov et al., 1997; Wallis & Galyov, 2000). In addition, evidence revealed that mutation of the virulence genes located on SPI-5 does not affect their ability in the development of systemic disease in mice (Wood et al., 1998). *sopD* is another effector protein involved in gastroenteritis and systemic infection in murine model. Deletion of *sopD* gene leads to reduction of fluid secretion and inflammatory responses during infection (Jones et al., 1998; Jiang et al., 2004), virulence of mutant is reduced but not abolished. Therefore, no significant difference in mice survivability between double knockout BD and parental *S.* Agona was observed in this study.

In recent years, different facultative and obligate anaerobic bacteria have been exploited as alternative tool in tumour treatment. These genetically engineered bacteria have been tested in experimental models and clinical trials due to their ability to overcome issues encounter with conventional therapy. *Salmonella* in particular has held great promise in showing anti-tumour properties and many researchers have used *Salmonella* strains to develop anti-tumour agents bearing different mutations. Researchers have revealed that the auxotrophic strain could colonize better in the tumour compartment. VNP20009, *Salmonella* auxotrophic for purine and adenine has been proven to effectively replicate in tumour tissue and cause tumour growth suppression in different murine tumour models (Luo et al., 2001). The approach taken in our study has applied the same concept by constructing the leucine and arginine auxotrophic *S.* Agona. Leu-Arg auxotrophic *S.* Agona showed tumour accumulation in ratio of 2000000:1 as compared to liver, indicating that genetically modified *S.* Agona auxotrophic strain prefer to colonize in tumour instead of normal tissues. This observation was in agreement with other studies where *Salmonella* prefer to colonize tumour at tumour-to-liver ratios of 250:1-10000:1 (Pawelek, Low & Bermudes, 1997; Zhao et al., 2004; Crull, Bumann & Weiss, 2011). Accumulation of bacteria in tumour region would induce apoptosis, eventually leads to tumour suppression (Li et al., 2012). In the present work, *Salmonella* enriched in tumour area and led to about 57–68 T/C% of tumour size *in vivo* and LA and BDLA strain showed better efficacy. Nevertheless, effectiveness of tumour growth suppression for the strains tested is not promising. Studies have shown that it is common that bacterial treatment in cancer therapy has to be used in conjunction with other chemotherapy drugs, or as delivery vector to carry a drug or pro-drug to the tumour microenvironment in order to enhance the anti-tumour efficacy (Leschner & Weiss, 2010). A study by Dang et al. (2004) showed dramatic tumour shrinkage when *C .novyi-NT* was used together with a specific microtubule-interacting drugs. Besides, oral delivery of *S.* Typhimurium VNP20009 together with 5-FU/Cisplatin in B16F10 melanoma mice demonstrated better anti-tumour efficacy than administrated VNP20009 alone (Chen et al., 2009). The YB1 strain, an engineered *Salmonella* that survives only in anaerobic conditions, was able to show total inhibition of tumour growth when administrated together with 5-FU (Yu et al., 2012). Synergistic effect could be obtained from combinational therapies since the chemotherapies might create a microenvironment which has more hypoxic and necrosis region, thus it is more suitable for bacteria to accumulate and replicate (Brown & Wilson, 2004; Chen et al., 2009). Hence, tumour suppression efficacy demonstrated in our study is not prominent since *S.* Agona was applied as monotherapy agent.

The relationship between tumour size and tumour stage remains controversial. As far as we know, no previous study have reported any *in vivo* correlation between tumour size and stage distribution. Hence, instead of focusing on tumour stage, tumour size is another concern that has been raised with regards to the tumour treatment using bacterial-based therapeutic agent. The effectiveness of bacterial-based anti-tumour therapy relies on the nutrient availability and oxygen levels in tumour microenvironment. It is not surprising that different size tumour may have different nutrient distribution and oxygen level. Previous study has demonstrated that *Salmonella* preferentially accumulate within necrotic regions of in vivo experimental model (Forbes et al., 2003). Necrotic areas in solid tumour contain high level of nutrients and lack immunologic surveillance, thus providing a suitable niche for *Salmonella* to survive and proliferate (Li et al., 2012). A large tumour is expected to have more areas of necrosis and hypoxia, thereby enhancing the potential of *Salmonella* to proliferate. The result obtained in our study was consistent with this hypothesis, where lower T/C% was achieved in larger tumours in the *in vivo* studies. Findings by Dang and colleagues support this observation as they showed that anti-tumour efficacy of *C. novyi-NT* was reduced with HCT116-induced tumour when the size was less than 100 mm^3^ (Dang et al., 2004). Nevertheless, the application of attenuated *S.* Agona in the treatment of small metastatic tumours requires further investigation. In addition, our finding also showed that multiple administration of treatment in tumour-bearing mice were not able to enhance the anti-tumour efficacy. It is possible that host innate and acquired immunity during primary infection of *Salmonella* play a central role in anti-tumour activity of *Salmonella*. Accordingly, it has been shown that the first administration of live *Salmonella* would prime the mouse immune system and the resultant immune cells contribute to the anti-tumour activity (Mastroeni & Menager, 2003; Xu et al., 1993). The presence of the anti-*Salmonella* antibody leads to the reduction of *Salmonella* invasiveness which eventually decreases the ability of *Salmonella* to target the tumour sites for the subsequent treatments (Lee et al., 2009).

## Conclusions

In conclusion, this study demonstrated the use of the group II intron in constructing quadruple knockout pathogens for the first time. The intron insertion in *S.* Agona was stable and no reversion to its parental phenotype was observed. This novel approach using group II intron mutagenesis would allow further improvement in bacteria for experimental and medical use. The findings in the mice survivability experiment were not able to meet our expectations for prolonging the mice survivability following infection. Yet quadruple knockout Δ*sopB*Δ*sopD*Δ*leuB*Δ*argD S.* Agona exhibited the lowest virulence in all tested parameters, suggesting that these four genes (*sopB*, *sopD*, *leuB*, *argD*) contribute to certain level of *Salmonella* virulence in systemic infection of the mouse model. Furthermore, present work demonstrated the use of auxotrophic *S.* Agona in CT-26 colon tumour treatment for the first time. Double and quadruple knockouts LA and BDLA were able to show lower T/C% value when compared to parental strain. Auxotroph *S.* Agona was attracted to nutrient-rich tumour resulting in tumour suppression. *sopB* and *sopD* virulence genes do not contribute to tumour fitness since double knockout BD showed almost similar T/C% with parental strain. Notably, the current study has shown that multiple administration of treatment was unable to enhance the anti-tumour efficacy significantly. Overall, the findings in this study help to gain an understanding of virulence and the role of metabolic gene deletions in fine-tuning *Salmonella* pathogenecity for successful tumour colonization and suppression.

##  Supplemental Information

10.7717/peerj.5989/supp-1 Figure S1 Potential intron insertion sites and primers designed for *leuB* and *argD* in *S.* Agona.

10.7717/peerj.5989/supp-2 Figure S2 PCR to validate the stability and purity of targeted clone (A) Sequences confirmation for successful knockout strain, Intron inserted genes were PCR amplified and sequenced using gene-specific primers (B&C) (A) Lane 1 and 2: Wild-type control template of *sopB* gene (1170); Lane 3: *sop* B mutated gene (2170 bp) in double knockout Δ*sopB*Δ*sopD*; Lane 4: *sopB* mutated gene (2170 bp) in quadruple knockout Δ*sopB* Δ*sopD*Δ*leuB*Δ*argD*; Lane 5 and 6: Wild type control template of *sopD* gene (310 bp); Lane 7: *sopD* mutated gene (1,310 bp) in double knockout Δ*sopB*Δ*sopD*; Lane 8: *sopD* mutated gene (1,310 bp) in quadruple knockout Δ*sopB* Δ*sopD* Δ*leuB* Δ*argD*; Lane 9 and 10: Wild-type control template of *leuB* gene (1,043 bp); Lane 11: *leuB* mutated gene (2,043 bp) in double knockout Δ*leuB*Δ*argD*; Lane 12: *leuB* mutated gene (2,043 bp) in quadruple knockout Δ*sopB*Δ*sopD*Δ*leuB*Δ*argD*; Lane 13 and 14: Wild-type control template of *argD* gene (887 bp); Lane 15: *argD* mutated gene (1,887 bp) in double knockout Δ*leuB* Δ*argD*; Lane 16: *argD* mutated gene (1,887 bp) in quadruple knockout Δ*sopB*Δ*sopD*Δ*leuB*Δ*argD*.

10.7717/peerj.5989/supp-3 Figure S3 Comparison analysis of bacterial load in spleen (A) and neutrophil (B) between tumour-free mice and CT-26 tumour bearing mice (A) Bacterial load in the spleen of tumour-free mice and CT-26 tumour-bearing mice. Mice were sacrificed and spleen was collected post treatment at indicated time point. Each bar represents the mean ±SEM of four mice per group. (*) *P* < 0.01 and (▾) *P* < 0.05 when compared with no tumour group. (B): Blood neutrophil percentage in tumour-free mice and CT-26 tumour-bearing mice. Mice were sacrificed and blood was collected post treatment at indicated time point. Each bar represents the mean ±SEM of four mice per group. (*) *P* < 0.01 and (▾) *P* < 0.05 when compared with no tumour group.

10.7717/peerj.5989/supp-4 Figure S4 Body weight of tumour bearing mice (A) and tumour growth curve (B) (A) Body weight of CT-26 tumour-bearing mice post treatment with wild-type and engineered strains of *S.* Agona. CT-26 mice were administrated intraperitoneally with different strains of *Salmonella* and the body weight of mice was taken every two days. No significant difference observed in the body weight throughout the study. (B) Tumour growth curve of CT-26 tumour-bearing mice post treatment with wild-type and engineered strains of *S.* Agona. CT-26 mice were administrated intraperitoneally with different strains of *Salmonella* and the tumour size was taken every two days.

10.7717/peerj.5989/supp-5 Figure S5 Raw data for bacterial growth curve

10.7717/peerj.5989/supp-6 Figure S6 Raw data for immunological profiles

10.7717/peerj.5989/supp-7 Data S1 Raw data of sequencing-knockout confirmation

10.7717/peerj.5989/supp-8 Data S2 Raw data of tumour growth suppression

10.7717/peerj.5989/supp-9 Data S3 Raw data of tumour bearing mice bacterial count

10.7717/peerj.5989/supp-10 Data S4 Raw data of tumour free mice bacterial count

## Additional Information and Declarations

### Data Availability

The following information was supplied regarding data availability: The raw data are provided in the Supplemental Files.
