# Supplementary figures and images for: Targeted inactivation of Salmonella Agona metabolic genes by group II introns and in vivo assessment of pathogenicity and anti-tumour activity in mouse model
Source: PeerJ. 2019 Jan 16;7:e5989. doi: 10.7717/peerj.5989 (PMC6339473; doi:10.7717/peerj.5989)

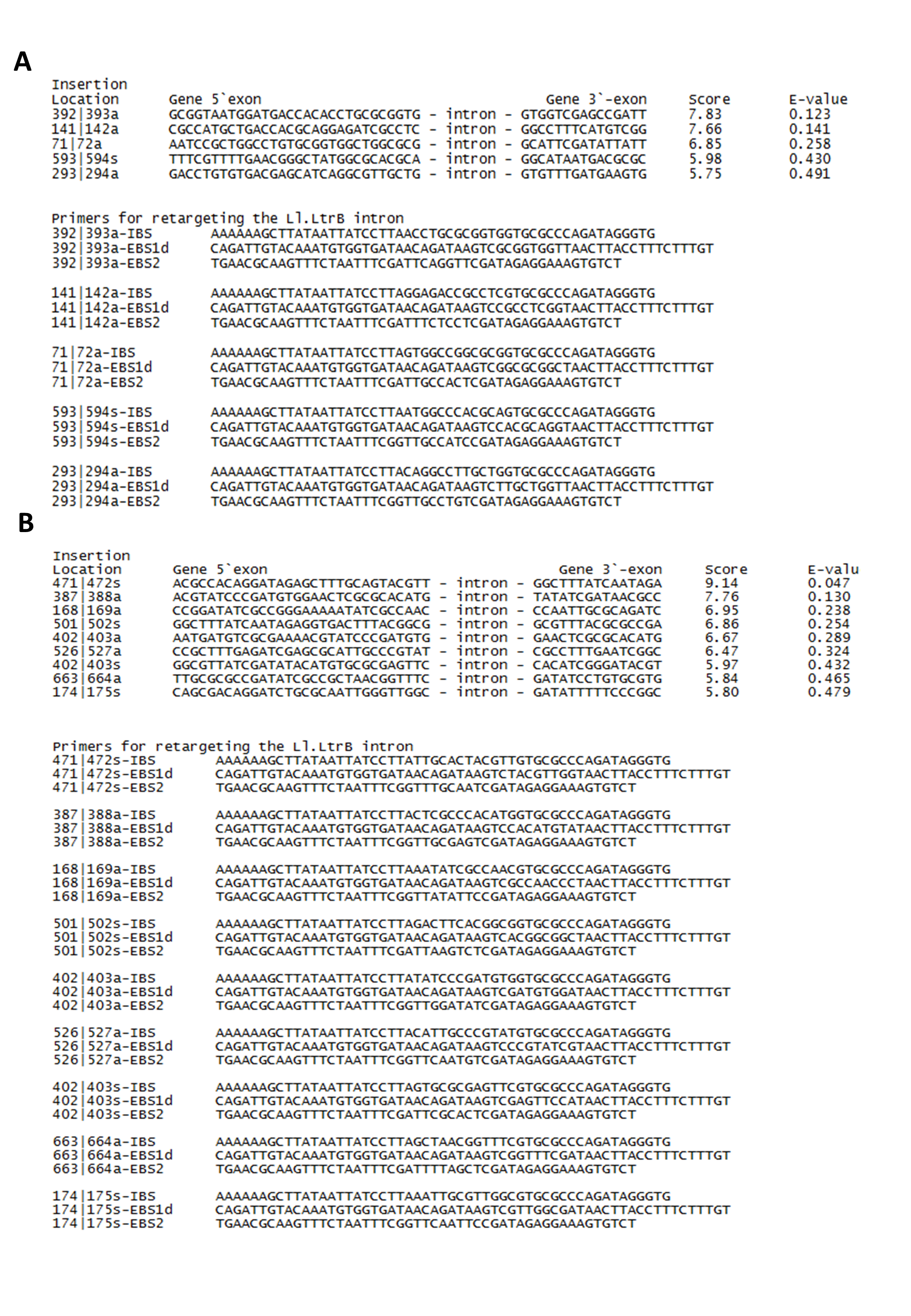

Supplement: Figure S1 [file peerj-07-5989-s001.png]

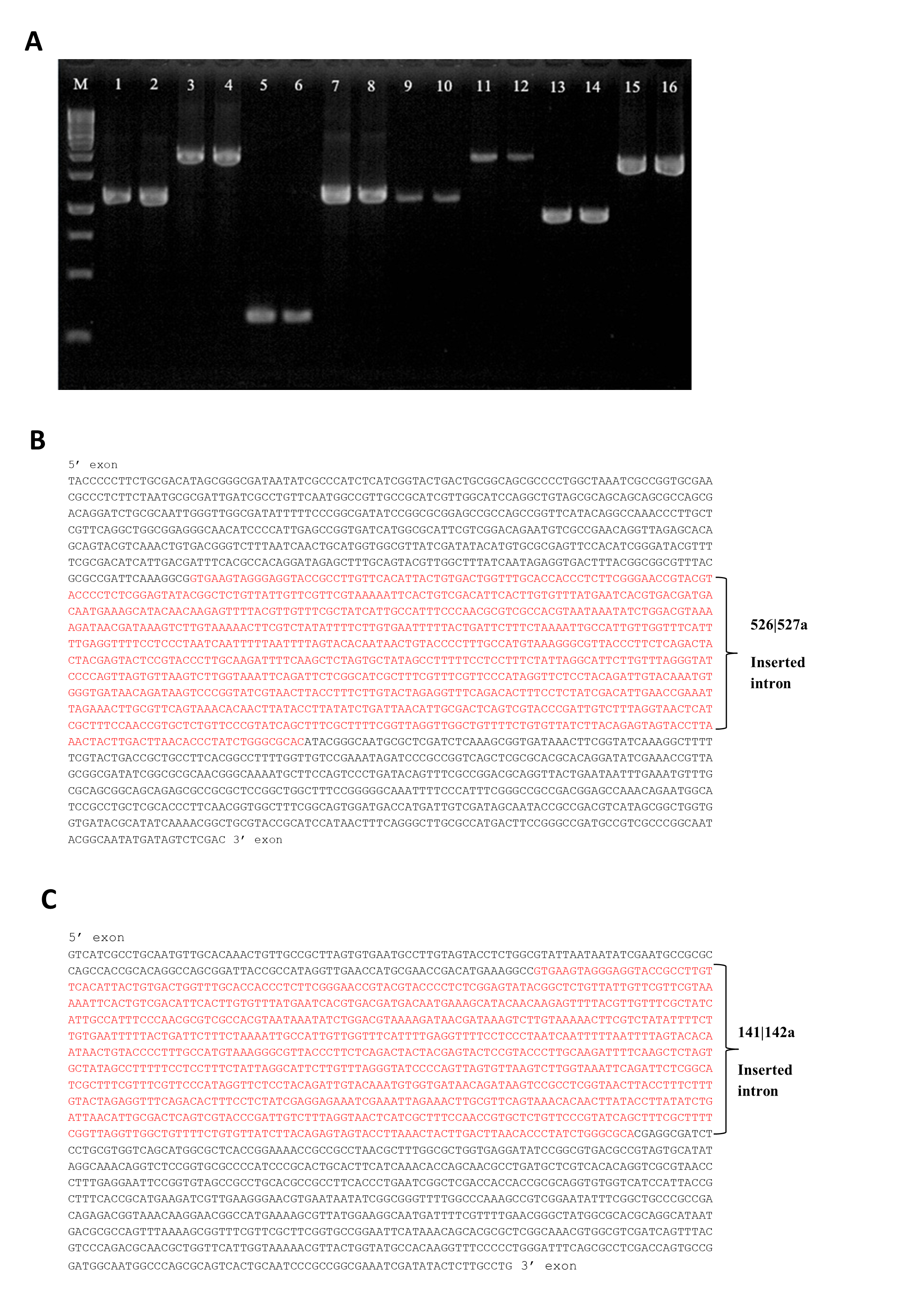

Supplement: Figure S2 — (A) Lane 1 and 2: Wild-type control template of sopB gene (1170); Lane 3: sop B mutated gene (2170 bp) in double knockout ΔsopBΔsopD; Lane 4: sopB mutated gene (2170 bp) in quadruple knockout ΔsopB ΔsopDΔleuBΔargD; Lane 5 and 6: Wild type control template of sopD gene (310 bp); Lane 7: sopD mutated gene (1,310 bp) in double knockout ΔsopBΔsopD; Lane 8: sopD mutated gene (1,310 bp) in quadruple knockout ΔsopB ΔsopD ΔleuB ΔargD; Lane 9 and 10: Wild-type control template of leuB gene (1,043 bp); Lane 11: leuB mutated gene (2,043 bp) in double knockout ΔleuBΔargD; Lane 12: leuB mutated gene (2,043 bp) in quadruple knockout ΔsopBΔsopDΔleuBΔargD; Lane 13 and 14: Wild-type control template of argD gene (887 bp); Lane 15: argD mutated gene (1,887 bp) in double knockout ΔleuB ΔargD; Lane 16: argD mutated gene (1,887 bp) in quadruple knockout ΔsopBΔsopDΔleuBΔargD. [file peerj-07-5989-s002.png]

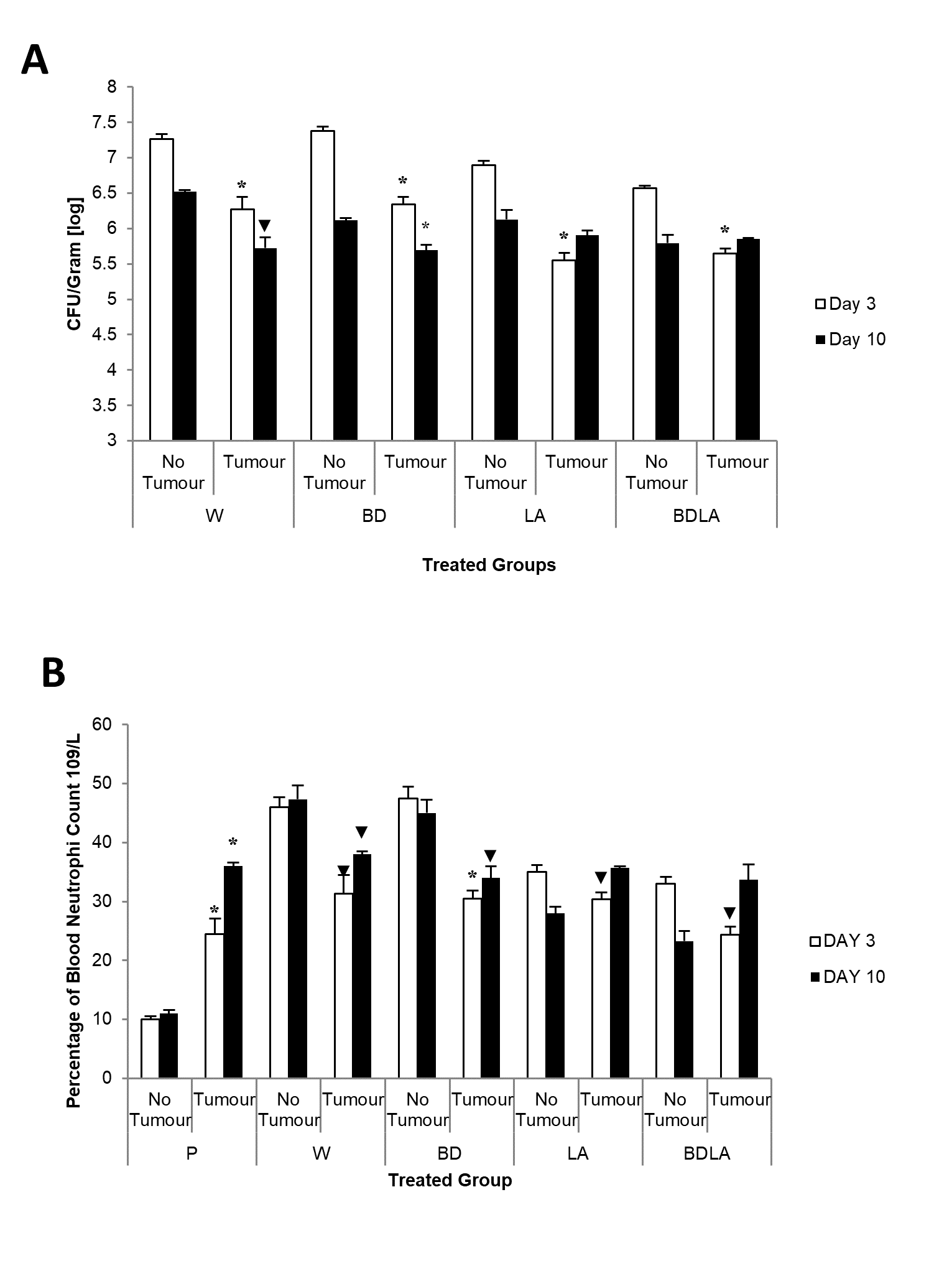

Supplement: Figure S3 — (A) Bacterial load in the spleen of tumour-free mice and CT-26 tumour-bearing mice. Mice were sacrificed and spleen was collected post treatment at indicated time point. Each bar represents the mean ±SEM of four mice per group. (*) P < 0.01 and (▾) P < 0.05 when compared with no tumour group. (B): Blood neutrophil percentage in tumour-free mice and CT-26 tumour-bearing mice. Mice were sacrificed and blood was collected post treatment at indicated time point. Each bar represents the mean ±SEM of four mice per group. (*) P < 0.01 and (▾) P < 0.05 when compared with no tumour group. [file peerj-07-5989-s003.png]

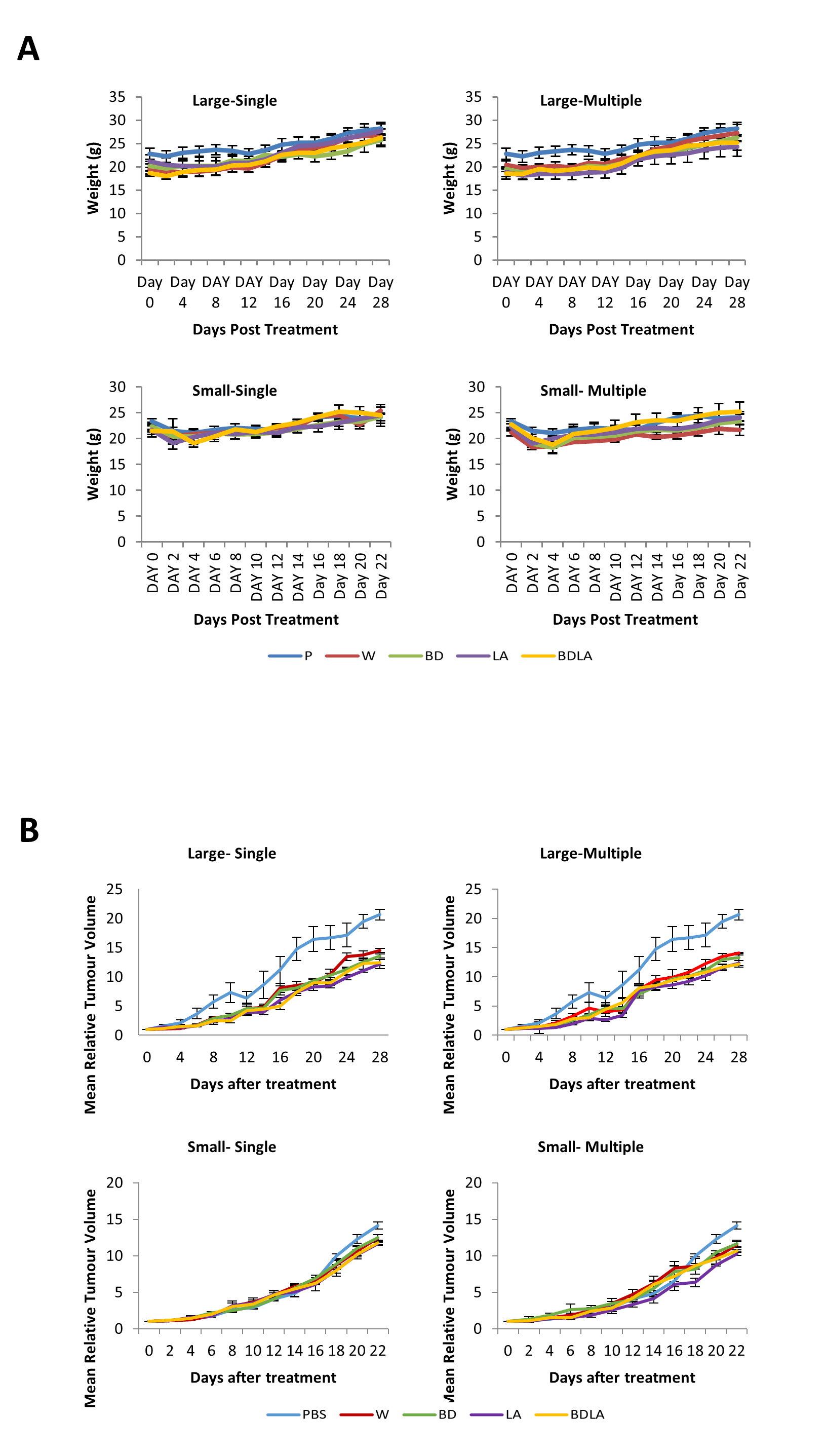

Supplement: Figure S4 — (A) Body weight of CT-26 tumour-bearing mice post treatment with wild-type and engineered strains of S. Agona. CT-26 mice were administrated intraperitoneally with different strains of Salmonella and the body weight of mice was taken every two days. No significant difference observed in the body weight throughout the study. (B) Tumour growth curve of CT-26 tumour-bearing mice post treatment with wild-type and engineered strains of S. Agona. CT-26 mice were administrated intraperitoneally with different strains of Salmonella and the tumour size was taken every two days. [file peerj-07-5989-s004.png]
