# Supplementary material for: Targeted inactivation of Salmonella Agona metabolic genes by group II introns and in vivo assessment of pathogenicity and anti-tumour activity in mouse model
Source: PeerJ. 2019 Jan 16;7:e5989. doi: 10.7717/peerj.5989 (PMC6339473; doi:10.7717/peerj.5989)
Supplement: Data S1 [file peerj-07-5989-s007.docx]

**Sequencing Data (Confirmation of *Salmonella* Knockouts)**

1. ***LeuB* Knockout**

**526|527a**

**Inserted intron**

5’ exon TACCCCCTTCTGCGACATAGCGGGCGATAATATCGCCCATCTCATCGGTACTGACTGCGGCAGCGCCCCTGGCTAAATCGCCGGTGCGAACGCCCTCTTCTAATGCGCGATTGATCGCCTGTTCAATGGCCGTTGCCGCATCGTTGGCATCCAGGCTGTAGCGCAGCAGCAGCGCCAGCGACAGGATCTGCGCAATTGGGTTGGCGATATTTTTCCCGGCGATATCCGGCGCGGAGCCGCCAGCCGGTTCATACAGGCCAAACCCTTGCTCGTTCAGGCTGGCGGAGGGCAACATCCCCATTGAGCCGGTGATCATGGCGCATTCGTCGGACAGAATGTCGCCGAACAGGTTAGAGCACAGCAGTACGTCAAACTGTGACGGGTCTTTAATCAACTGCATGGTGGCGTTATCGATATACATGTGCGCGAGTTCCACATCGGGATACGTTTTCGCGACATCATTGACGATTTCACGCCACAGGATAGAGCTTTGCAGTACGTTGGCTTTATCAATAGAGGTGACTTTACGGCGGCGTTTACGCGCCGATTCAAAGGCGGTGAAGTAGGGAGGTACCGCCTTGTTCACATTACTGTGACTGGTTTGCACCACCCTCTTCGGGAACCGTACGTACCCCTCTCGGAGTATACGGCTCTGTTATTGTTCGTTCGTAAAAATTCACTGTCGACATTCACTTGTGTTTATGAATCACGTGACGATGACAATGAAAGCATACAACAAGAGTTTTACGTTGTTTCGCTATCATTGCCATTTCCCAACGCGTCGCCACGTAATAAATATCTGGACGTAAAAGATAACGATAAAGTCTTGTAAAAACTTCGTCTATATTTTCTTGTGAATTTTTACTGATTCTTTCTAAAATTGCCATTGTTGGTTTCATTTTGAGGTTTTCCTCCCTAATCAATTTTTAATTTTAGTACACAATAACTGTACCCCTTTGCCATGTAAAGGGCGTTACCCTTCTCAGACTACTACGAGTACTCCGTACCCTTGCAAGATTTTCAAGCTCTAGTGCTATAGCCTTTTTCCTCCTTTCTATTAGGCATTCTTGTTTAGGGTATCCCCAGTTAGTGTTAAGTCTTGGTAAATTCAGATTCTCGGCATCGCTTTCGTTTCGTTCCCATAGGTTCTCCTACAGATTGTACAAATGTGGGTGATAACAGATAAGTCCCGGTATCGTAACTTACCTTTCTTGTACTAGAGGTTTCAGACACTTTCCTCTATCGACATTGAACCGAAATTAGAAACTTGCGTTCAGTAAACACAACTTATACCTTATATCTGATTAACATTGCGACTCAGTCGTACCCGATTGTCTTTAGGTAACTCATCGCTTTCCAACCGTGCTCTGTTCCCGTATCAGCTTTCGCTTTTCGGTTAGGTTGGCTGTTTTCTGTGTTATCTTACAGAGTAGTACCTTAAACTACTTGACTTAACACCCTATCTGGGCGCACATACGGGCAATGCGCTCGATCTCAAAGCGGTGATAAACTTCGGTATCAAAGGCTTTTTCGTACTGACCGCTGCCTTCACGGCCTTTTGGTTGTCCGAAATAGATCCCGCCGGTCAGCTCGCGCACGCACAGGATATCGAAACCGTTAGCGGCGATATCGGCGCGCAACGGGCAAAATGCTTCCAGTCCCTGATACAGTTTCGCCGGACGCAGGTTACTGAATAATTTGAAATGTTTGCGCAGCGGCAGCAGAGCGCCGCGCTCCGGCTGGCTTTCCGGGGGCAAATTTTCCCATTTCGGGCCGCCGACGGAGCCAAACAGAATGGCATCCGCCTGCTCGCACCCTTCAACGGTGGCTTTCGGCAGTGGATGACCATGATTGTCGATAGCAATACCGCCGACGTCATAGCGGCTGGTGGTGATACGCATATCAAAACGGCTGCGTACCGCATCCATAACTTTCAGGGCTTGCGCCATGACTTCCGGGCCGATGCCGTCGCCCGGCAATACGGCAATATGATAGTCTCGAC 3’ exon

1. ***ArgD* Knockout**

**141|142a**

**Inserted intron**

5’ exon GTCATCGCCTGCAATGTTGCACAAACTGTTGCCGCTTAGTGTGAATGCCTTGTAGTACCTCTGGCGTATTAATAATATCGAATGCCGCGCCAGCCACCGCACAGGCCAGCGGATTACCGCCATAGGTTGAACCATGCGAACCGACATGAAAGGCCGTGAAGTAGGGAGGTACCGCCTTGTTCACATTACTGTGACTGGTTTGCACCACCCTCTTCGGGAACCGTACGTACCCCTCTCGGAGTATACGGCTCTGTTATTGTTCGTTCGTAAAAATTCACTGTCGACATTCACTTGTGTTTATGAATCACGTGACGATGACAATGAAAGCATACAACAAGAGTTTTACGTTGTTTCGCTATCATTGCCATTTCCCAACGCGTCGCCACGTAATAAATATCTGGACGTAAAAGATAACGATAAAGTCTTGTAAAAACTTCGTCTATATTTTCTTGTGAATTTTTACTGATTCTTTCTAAAATTGCCATTGTTGGTTTCATTTTGAGGTTTTCCTCCCTAATCAATTTTTAATTTTAGTACACAATAACTGTACCCCTTTGCCATGTAAAGGGCGTTACCCTTCTCAGACTACTACGAGTACTCCGTACCCTTGCAAGATTTTCAAGCTCTAGTGCTATAGCCTTTTTCCTCCTTTCTATTAGGCATTCTTGTTTAGGGTATCCCCAGTTAGTGTTAAGTCTTGGTAAATTCAGATTCTCGGCATCGCTTTCGTTTCGTTCCCATAGGTTCTCCTACAGATTGTACAAATGTGGTGATAACAGATAAGTCCGCCTCGGTAACTTACCTTTCTTTGTACTAGAGGTTTCAGACACTTTCCTCTATCGAGGAGAAATCGAAATTAGAAACTTGCGTTCAGTAAACACAACTTATACCTTATATCTGATTAACATTGCGACTCAGTCGTACCCGATTGTCTTTAGGTAACTCATCGCTTTCCAACCGTGCTCTGTTCCCGTATCAGCTTTCGCTTTTCGGTTAGGTTGGCTGTTTTCTGTGTTATCTTACAGAGTAGTACCTTAAACTACTTGACTTAACACCCTATCTGGGCGCACGAGGCGATCTCCTGCGTGGTCAGCATGGCGCTCACCGGAAAACCGCCGCCTAACGCTTTGGCGCTGGTGAGGATATCCGGCGTGACGCCGTAGTGCATATAGGCAAACAGGTCTCCGGTGCGCCCCATCCCGCACTGCACTTCATCAAACACCAGCAACGCCTGATGCTCGTCACACAGGTCGCGTAACCCTTTGAGGAATTCCGGTGTAGCCGCCTGCACGCCGCCTTCACCCTGAATCGGCTCGACCACCACCGCGCAGGTGTGGTCATCCATTACCGCTTTCACCGCATGAAGATCGTTGAAGGGAACGTGAATAATATCGGCGGGTTTTGGCCCAAAGCCGTCGGAATATTTCGGCTGCCCGCCGACAGAGACGGTAAACAAGGAACGGCCATGAAAAGCGTTATGGAAGGCAATGATTTTCGTTTTGAACGGGCTATGGCGCACGCAGGCATAATGACGCGCCAGTTTAAAAGCGGTTTCGTTCGCTTCGGTGCCGGAATTCATAAACAGCACGCGCTCGGCAAACGTGGCGTCGATCAGTTTACGTCCCAGACGCAACGCTGGTTCATTGGTAAAAACGTTACTGGTATGCCACAAGGTTTCCCCCTGGGATTTCAGCGCCTCGACCAGTGCCGGATGGCAATGGCCCAGCGCAGTCACTGCAATCCCGCCGGCGAAATCGATATACTCTTGCCTG 3’ exon
